# Multi-Response Optimization of FDM Process Parameters for Enhanced Mechanical Performance and Reduced Weight of PLA and PLA-CF 3D-Printed Parts

**DOI:** 10.3390/polym18182268

**Published:** 2026-09-17

**Authors:** Mustafa Saleh

**Affiliations:** Industrial Engineering Department, College of Engineering, King Saud University, P.O. Box 800, Riyadh 11421, Saudi Arabia; msaleh3@ksu.edu.sa

**Keywords:** FDM, extrusion parameters, optimization, PLA, carbon fiber-reinforced PLA, tensile properties, material consumption, RSM

## Abstract

This work investigates the influence of fused deposition modeling (FDM) parameters on the tensile properties and material consumption of FDM-printed samples. The study focused on extrusion-related parameters that directly govern material deposition during FDM printing, including extrusion temperature (ET), layer thickness (LT), and extrusion multiplier (EM). The combined effect of these parameters on the properties of FDM-printed parts has not been sufficiently investigated, particularly for polymer composites. Therefore, neat polylactic acid (PLA) and carbon fiber-reinforced PLA (PLA-CF) were considered. Standard tensile samples were used to evaluate the effect of ET, LT, and EM on the modulus of elasticity (E), yield strength (yield), ultimate tensile strength (UTS), and sample weight. Statistical analysis was conducted based on the response surface methodology (RSM). Desirability analysis was used as a multi-objective optimization method to maximize tensile properties and minimize material consumption. Fractographic analysis was performed using scanning electron microscopy. Statistical results showed that EM and ET significantly influenced E, yield strength, and UTS, with EM being the most influential factor, contributing 75.47–90.50% to the responses in both materials. EM had a greater influence on the tensile properties of neat PLA than PLA-CF, whereas ET showed the opposite trend, with a stronger influence on PLA-CF than on neat PLA. Increasing EM enhanced tensile performance by shifting the failure mechanism from highly raster-dominated/interfacial fracture occurring along the print boundaries at low EM (96%) to bulk-material-dominated fracture at higher EM (104%). The higher prevalence of pores and voids in PLA-CF resulted in lower yield strength and UTS than neat PLA, highlighting the need to optimize FDM parameters for composite materials. The optimal settings (230 °C ET, 0.15 mm LT, and 104% EM) simultaneously maximized E (PLA: 3.583 GPa; PLA-CF: 4.507 GPa), yield (PLA: 51.23 MPa; PLA-CF: 41.30 MPa), and UTS (PLA: 55.13 MPa; PLA-CF: 47.57 MPa) while minimizing specimen weight (PLA: 5.907 g; PLA-CF: 5.728 g). However, the sensitivity analysis provided additional insight by showing that a slight increase in EM from 104% to 106% further improved the tensile properties of PLA-CF, whereas 104% remained the optimal EM for neat PLA.

## 1. Introduction

Additive manufacturing (AM) technologies are free-form fabrication processes that convert a CAD model into a 3D part by building it layer by layer. Fused deposition modeling (FDM), known as the material extrusion AM process, is the most commonly used AM process for producing prototypes and functional parts [1]. The FDM process is versatile, simple, and affordable. Polymer-based materials, including single polymers, blends, and composites, can be used in FDM printing. Despite FDM’s popularity, issues such as weak interlayer/intra-layer bonding, low density, and anisotropy limit its widespread use [2]. The geometric, mechanical, surface, and functional quality characteristics of FDM-printed parts are highly influenced by the printing parameters. Moreover, polymers generally exhibit lower mechanical performance than other engineering materials, such as metals. Therefore, filler materials, including fibers and particles, are incorporated to reinforce polymers and enhance their mechanical performance [3,4], as well as other properties, such as thermal and electrical performance [5,6]. However, incorporating such reinforcements into polymers alters material behavior during extrusion due to the interaction between the polymer matrix and the filler, thereby influencing print quality [7]. For instance, FDM printing of composite materials is generally attributed to the formation of voids due to increased viscosity [8]. The formation of such voids deteriorates the mechanical performance of FDM-printed parts and can be reduced by optimizing the FDM printing parameters [9]. Consequently, the proper selection of FDM printing parameters is essential to achieve the desired performance [10].

Numerous studies have been conducted to optimize various FDM parameters, with relatively less attention paid to the extrusion multiplier in combination with other FDM parameters, such as extrusion temperature and layer thickness, which directly control material deposition during printing. Luis-Pérez and Esquíroz-Cabodevilla [11] statistically studied the effect of extrusion temperature (200–210 °C), extrusion multiplier (90 and 100%), printing speed (30–50 mm/s), layer thickness (0.1–0.2 mm), and nozzle diameter (0.25–0.8 mm) on the surface roughness, density, and tensile strength. Prediction modeling was developed by ANVIS and integrated with particle swarm optimization for multi-objective optimization of PLA screw prototypes. Findings showed that the optimal settings of 210 °C extrusion temperature, 90% extrusion multiplier, 30 mm/s printing speed, 0.1 mm layer thickness, and 0.25 mm nozzle diameter achieved the best results. Lendvai et al. [12] examined the effect of modifying the extrusion multiplier (97–105%) on the geometric, mechanical (tensile, flexural, and Charpy impact), and thermal conductivity characteristics of FDM-printed PLA parts. The density, mechanical properties, and thermal conductivity were improved until the extrusion multiplier reached 103%, according to the results. However, after 103%, the geometrical precision diminished due to excessive material being compressed. Moreover, the Charpy impact strength and flexural strength began to diminish after 103% (at 105%). Dusanapudi and Krupakaran [9] evaluated the impact of FDM factors, such as extrusion multiplier, temperature, and layer thickness, on the tensile and compressive strengths of ABS parts using Taguchi design. Also, gray relational analysis was integrated with a genetic algorithm to optimize the mentioned parameters. The density and tensile strength increased linearly with increasing extrusion multiplier (85–100%). The optimal parameters were determined to be a 100% extrusion multiplier, a temperature of 240 °C, and a layer thickness of 0.1 mm. Lambiase et al. [13] studied the effect of the extrusion multiplier (99–104%) on the compression and indentation yield strength of PLA-FDM-printed cylinder samples. The findings indicated that the yield strengths for compression and indentation increased linearly with part density, which also increased linearly with the extrusion multiplier. Lambiase et al. [14] utilized a full factorial design to examine the effects of raster angle, extrusion multiplier, and printing speed on the interlayer adhesion and fracture toughness of FDM-printed PLA. Findings showed that the considered parameters significantly influence interlayer adhesion. The increased material flow led to the formation of a crystalline phase post-deposition. Samples with an extrusion multiplier of 100% showed improved fracture toughness compared to those with an extrusion multiplier of 104%, which had poor adhesion attributed to the higher crystallinity associated with increased material flow (104%). The ±45° raster direction was found to be more reliable than 0/90°, which necessitated a higher flow rate (104%) to achieve satisfactory interlayer bonding. Bhaskar and Mohaghegh [15] studied the effect of FDM parameters, including extrusion temperature (180–210 °C) and material extrusion rate (70–160%), on the mass, dimensional accuracy, surface roughness, and tensile properties of PLA and PLA reinforced with graphene nanoplatelets (PLA/GNP). Changes in material extrusion rate have a higher impact on PLA/GNP than on PLA. The results showed that the mass of PLA samples did not increase over the considered range of temperatures or extrusion rates compared to the masses measured by the slicer (Cura 4.7.1 software). However, nearly all of the PLA/GNP samples showed a significant increase in mass with increasing extrusion rate. Both extrusion temperature and material extrusion rate significantly influenced the dimensional accuracy, surface roughness, and tensile properties with different patterns. Mukka et al. [16] studied the influence of layer thickness, extrusion multiplier, printing speed, and extrusion temperature on the surface finish of curved parts made from PLA based on Taguchi design and reported that extrusion multiplier showed the highest contribution (84.30%) to the surface roughness of the curved parts, followed by extrusion temperature, speed, and finally layer thickness. Ghorbani et al. [2] studied the influence of extrusion multiplier (100–120%) on the structure and interlayer strength of FFF parts made from ABS. Results showed that increasing the extrusion multiplier enhanced the tensile modulus and strength, with a more pronounced effect on samples printed in Z-orientation, yielding a ~50% increase in tensile strength compared to the predefined setting (e.g., 100% extrusion multiplier). By increasing the extrusion multiplier, void-free parts with nearly isotropic strength can be produced, which implies that mechanical anisotropy is not inherent to FDM.

The findings of these studies demonstrate that extrusion-related parameters, particularly the extrusion multiplier, can substantially affect the mechanical properties, surface quality, geometrical accuracy, and material consumption of FDM-printed parts. Although these studies demonstrate the importance of extrusion-related parameters, their findings also indicate that the effect of the extrusion multiplier depends on the material, parameter range, and other FDM processing conditions. Few researchers have explored the combination of extrusion-related parameters, including extrusion temperature, extrusion multiplier, and layer thickness [9,11,16]. Most existing studies address only the extrusion multiplier in single polymers such as ABS [2,9] and PLA [11,12,13,14,16], with limited work on polymer composites, as in FDM [15] and fused granular fabrication printer [17]. The influence of these printing parameters on polymer composites may differ from that on neat polymers due to variations in their thermal properties and matrix-filler interactions. FDM printing of polymer composites is subject to thermal processes that may introduce structural discontinuities, such as pores or voids, which are more pronounced with microfillers than nanofillers [17]. Bhaskar and Mohaghegh [15] reported that PLA reinforced with graphene is more influenced by varying material extrusion rate than pure PLA. The quality of FDM-printed parts is governed by coupled interactions between material behavior and processing conditions [18]. Furthermore, the literature review highlighted inconsistencies in the reported findings. For instance, [2,15] reported that over-extrusion reduces voids and interlayer gaps, thereby improving strength. In contrast, [6,14] reported that over-extrusion diminishes mechanical properties due to excessive material compression [6] and poor adhesion attributed to higher crystallinity [14]. These differences further indicate that the effect of extrusion-related parameters cannot be generalized across different materials and processing conditions. Therefore, a systematic study is needed to investigate how the individual and combined effects of the three extrusion-related parameters, including extrusion temperature, extrusion multiplier, and layer thickness, influence mechanical performance and material consumption across both neat polymers and polymer composites.

The present study investigates the influence of extrusion temperature, extrusion multiplier, and layer thickness on tensile properties, namely Young’s modulus, yield strength, and ultimate tensile strength, in addition to material consumption. Two materials were considered: neat PLA and a carbon fiber-reinforced polymer composite (PLA-CF). The work was conducted using response surface methodology, which can investigate the single and combined effects of FDM printing parameters and develop second-order prediction models. Desirability analysis was used as a multi-objective optimization method to maximize tensile properties and minimize material consumption. A sensitivity analysis was subsequently conducted to investigate the effect of increasing the extrusion multiplier beyond the RSM range. Furthermore, the morphology of the fractured tensile cross-sections was analyzed using scanning electron microscopy (SEM).

## 2. Materials and Methods

### 2.1. Materials

Two materials in the form of filaments with a diameter of 1.75 mm were used in this study: (1) polylactic acid (PLA) filament, supplied by Prusa, Prague, Czech Republic, and (2) carbon fiber-reinforced PLA (PLA-CF) filament containing 15 wt.% short carbon fibers, supplied by 3DXTech, Grand Rapids, MI, USA. Both materials use NatureWorks Ingeo 4043D PLA biopolymer (NatureWorks LLC, Blair, NE, USA) as the PLA grade. The materials were used as received from the supplier without additional drying treatment. The filaments were stored in an air-conditioned laboratory environment at approximately room temperature. The characteristics of PLA and PLA-CF materials are presented in Table 1, as reported in the manufacturer’s datasheet.

**Figure 1:**
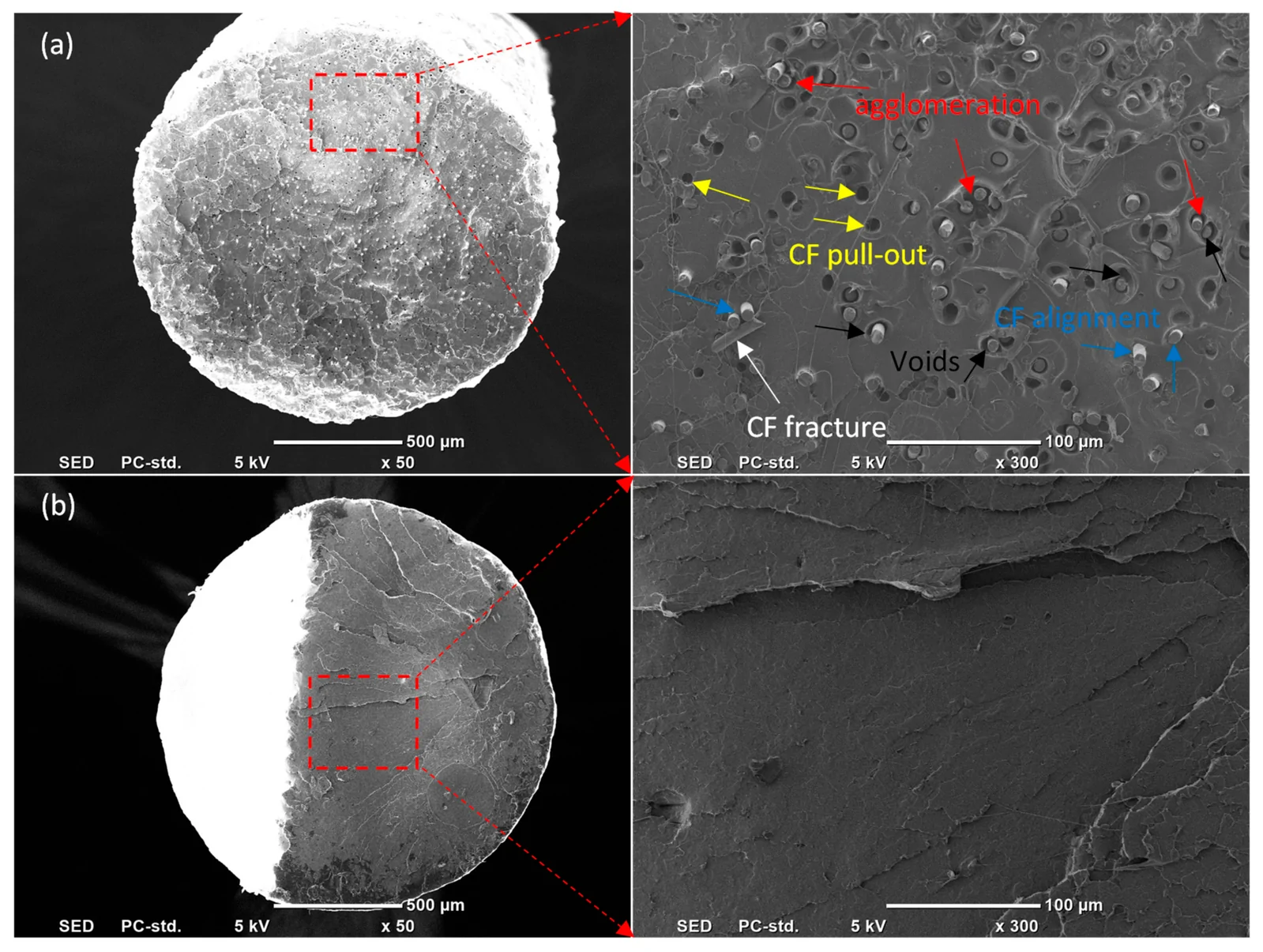
SEM images showing the filaments’ cross-sectional views of: (**a**) PLA-CF and (**b**) PLA.

The diameters of the PLA and PLA-CF filaments were measured using a digital micrometer (Preisser 0912501, Preisser, Gammertingen, Germany) at 10 different locations, and the average values are reported in Table 2. The thermal behavior of PLA and PLA-CF filaments was characterized using differential scanning calorimetry (DSC) with an LR-STA-200 (Lonory, Dongguan, China). The glass transition (Tg), cold crystallization (Tcc), and melting (Tm) temperatures were determined by heating the samples from ambient temperature to 200 °C at a rate of 10 °C/min using an aluminum crucible under a nitrogen (N_2_) atmosphere. The Tg, Tcc, and Tm of the PLA and PLA-CF filaments are reported in Table 2. The cross-sectional morphology of the PLA and PLA-CF filaments was examined using scanning electron microscopy (SEM) (JEOL JCM 6000 plus, Tokyo, Japan). The filament samples were first immersed in liquid nitrogen for 10 min and then cryogenically fractured, followed by platinum coating using an Auto Fine Coater (JEC-3000FC, Tokyo, Japan). Figure 1a presents the SEM cross-sectional morphology of the PLA-CF filament, showing the distribution and preferential alignment of CFs along the filament’s longitudinal direction. The SEM image also shows CF pull-out and breakage, CF agglomeration, and voids/pores surrounding the CF. Figure 1b shows the PLA filament with a relatively homogeneous and compact structure, without the pores observed in the PLA-CF filament.

### 2.2. FDM Printing and Experimental Design

The FDM process parameters considered in this study are extrusion temperature (ET), layer thickness (LT), and extrusion multiplier (EM). ET represents the filament temperature in the extruder before it exits the nozzle. LT denotes the thickness of a single deposited layer along the Z-axis. EM is a parameter that governs the material flow rate, i.e., the amount of material deposited during FDM printing. The effects of ET, LT, and EM on the mechanical and weight characteristics were evaluated using statistical analysis. Response surface methodology (RSM) with a face-centered central composite design, a design of experiments (DOE) approach, was employed. With three variables (ET, LT, and EM), the RSM design generated 20 experimental runs. Samples (i.e., standard tensile specimens) were replicated three times for each of the 20 RSM runs, resulting in a total of 60 samples for each material (PLA and PLA-CF). The three replicate specimens for each RSM run were FDM-printed in a single print job under the same processing conditions. The 20 RSM runs were randomly FDM-printed and tested according to the randomized order generated by the statistical software. The statistical significance of the variables and their interactions was evaluated using analysis of variance (ANOVA) at the 95% confidence level. Regression models were developed to predict responses. To enhance the predictability of the regression models, reduced ANOVA tables were developed using a stepwise elimination algorithm. The prediction models were then employed to optimize the ET, LT, and EM settings, with the objective of simultaneously maximizing the tensile modulus, yield strength, and ultimate tensile strength while minimizing specimen weight. The multi-objective optimization was conducted using the desirability approach, with equal importance assigned to all four responses. Statistical analysis, predictive modeling, and optimization were performed using Minitab 18.

**Figure 2:**
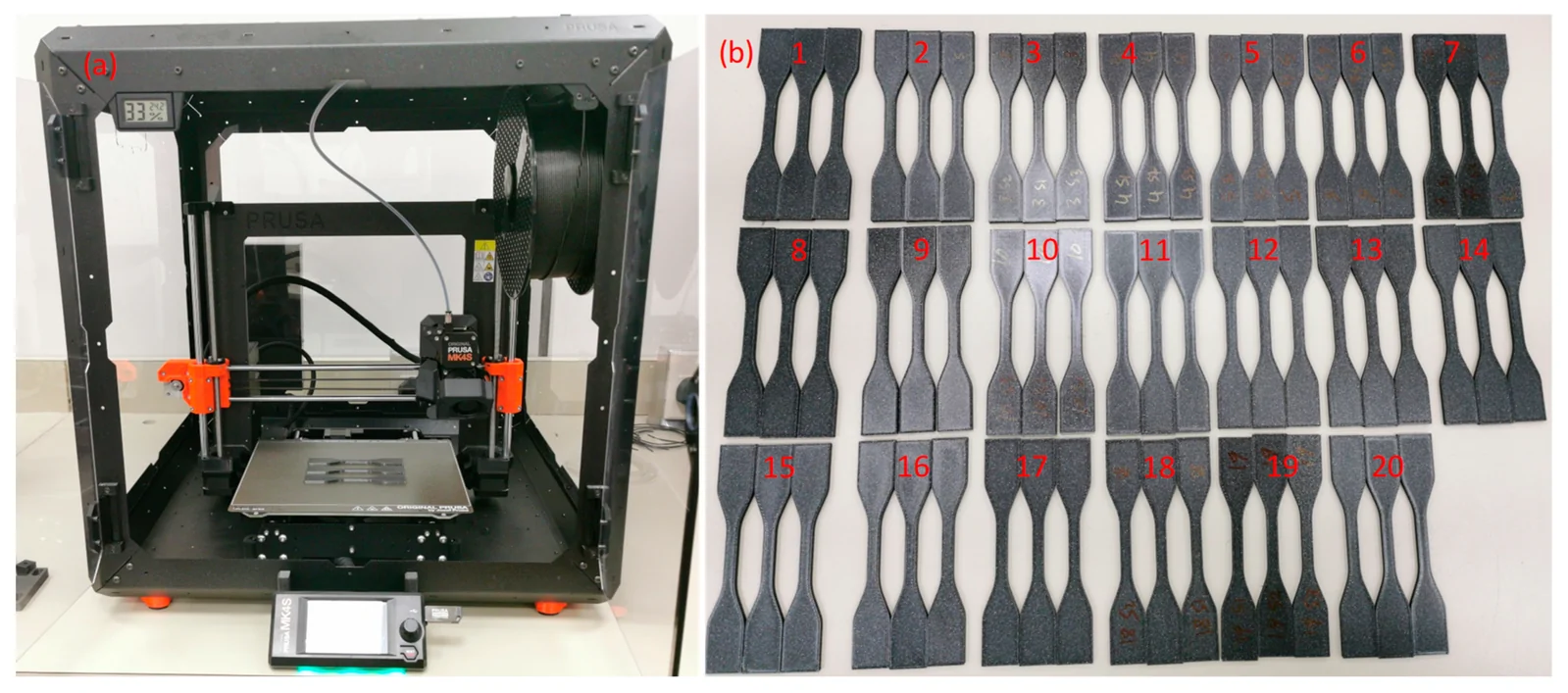
(**a**) FDM printing and (**b**) FDM-printed tensile samples (i.e., PLA samples). The numbers 1 to 20 represent the corresponding 20 printed samples produced according to the RSM runs.

The levels of the considered FDM parameters (ET, LT, and EM) are reported in Table 3. A Prusa FDM printer (Prusa MK4S, Prague, Czech Republic) was used to fabricate tensile-testing specimens. The specimens were fabricated using a 0.4 mm nozzle, 100% rectilinear infill, two perimetral shells (0.45 mm raster width), a printing speed of 80 mm/s, bed temperature of 60 °C, a raster angle of ±45°, and disabled fan cooling speed for the 1st layer and full (100%) fan speed from the 3rd layer. Figure 2 shows FDM printing and examples of FDM-printed tensile samples.

### 2.3. Mechanical Characterization

**Figure 3:**
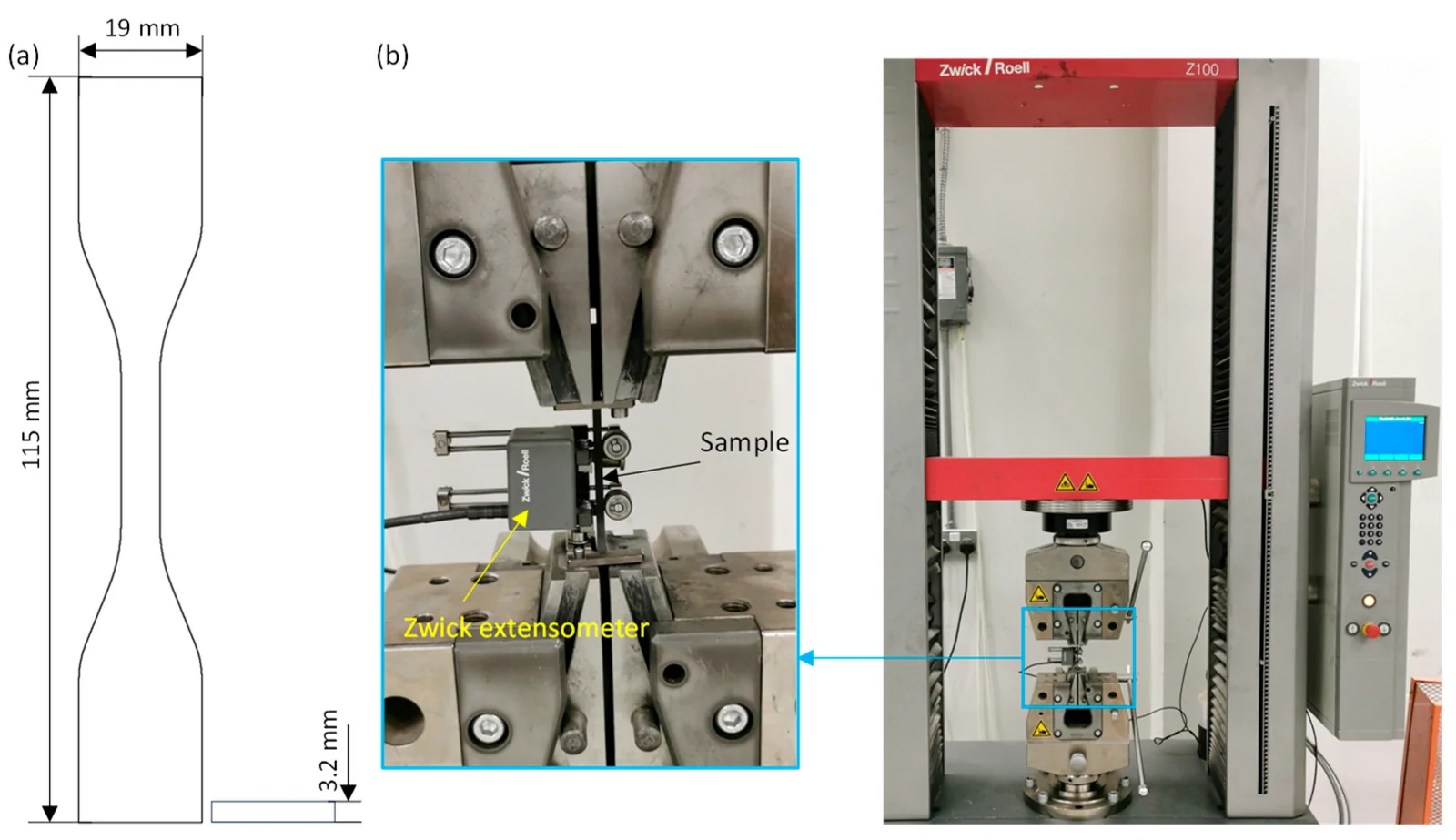
(**a**) ASTM D638 Type IV tensile test specimen design and (**b**) tensile testing setup.

The influence of the FDM parameters ET, LT, and EM on the mechanical performance of FDM-printed parts was assessed through uniaxial tensile testing. Tensile specimens conforming to ASTM D638-14 [21], Type IV, (see Figure 3a) were designed and printed. The tensile specimens were kept in the same air-conditioned laboratory environment for 10 days prior to tensile testing. The tests were performed on a Zwick Roell Z100 universal testing machine (ZwickRoell GmbH & Co. KG, Ulm, Germany) (see Figure 3b) at a constant crosshead speed of 5 mm/min until fracture. The following tensile properties were considered: modulus of elasticity (E), yield strength at 0.2% offset (Yield), and ultimate tensile strength (UTS). A Zwick extensometer was used to accurately determine E and Yield. As mentioned earlier, three specimens were tested for each RSM run, and the average values were used for statistical analysis.

### 2.4. Metrology Characterization

**Figure 4:**
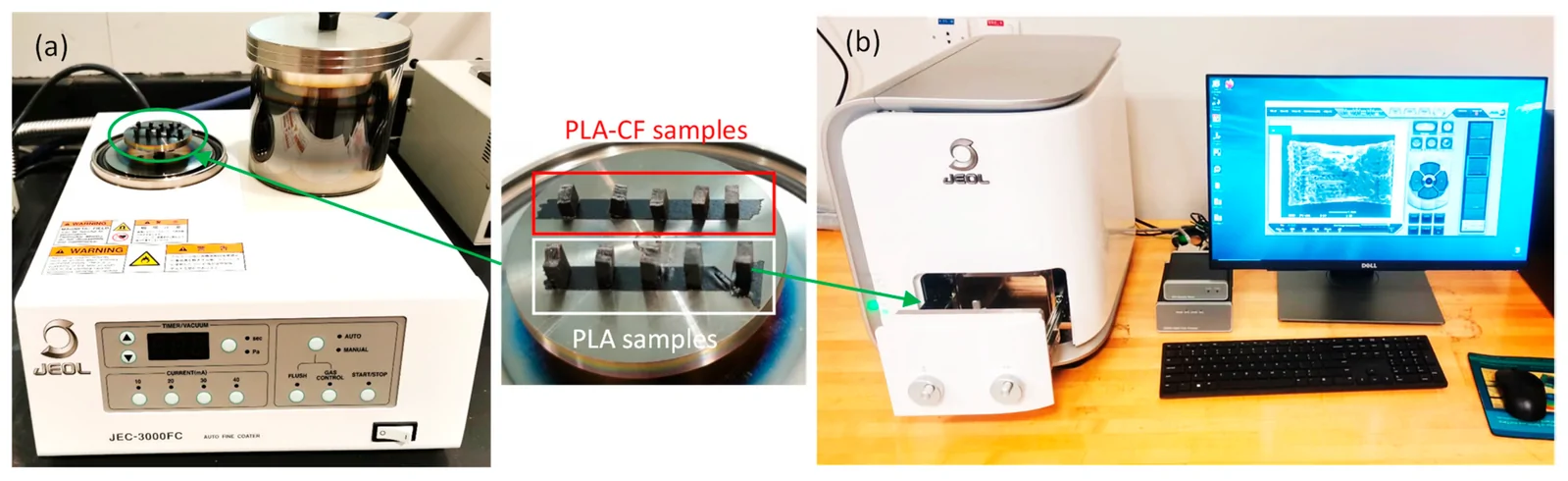
Morphological characterization process: (**a**) sample coating and (**b**) SEM analysis.

The weight of the FDM-printed tensile samples was measured using a precision analytical balance (0.01 mg) (AUW220D, Shimadzu, Kyoto, Japan). The morphology of the fracture surfaces of the tensile specimens was characterized using scanning electron microscopy (SEM) (JEOL JCM 6000 plus, Tokyo, Japan) at an accelerating voltage of 5 kV and a working distance of 19 mm, using a secondary electron detector (SED), over a magnification range of 20× to 300×. Prior to SEM imaging, the fracture surfaces were platinum-coated by sputtering for 60 s at 30 mA using an Auto Fine Coater (JEC-3000FC, Tokyo, Japan) to enhance imaging quality; see Figure 4. The entire fracture surface was first examined at low magnification (20×), and representative regions exhibiting characteristic fracture features were subsequently selected and examined at higher magnifications to provide detailed morphological observations. The width and thickness of the tensile specimens at the gauge section were measured at three locations using a digital micrometer (Preisser 0912501) with a measurement range of 0–25 mm, a resolution of 0.001 mm, and an accuracy of ±0.006 mm. These measured dimensions were used to determine the actual cross-sectional area of each specimen and, accordingly, to calculate the tensile properties.

### 2.5. Sensitivity Analysis

Following the completion of the RSM experiments and identification of the optimized processing condition, a sensitivity analysis was conducted to investigate the effect of further increasing the EM beyond the range considered in the RSM design. The optimized EM of 104% was used as the reference condition, and additional EM levels of 106%, 110%, and 115% were subsequently investigated based on the trends observed in the RSM results. During these experiments, the optimized ET and layer thickness LT were kept constant, while only EM was increased. These additional experiments were conducted independently of the original RSM design to provide further insight into the effect of increasing EM on the considered responses.

## 3. Results

**Figure 5:**
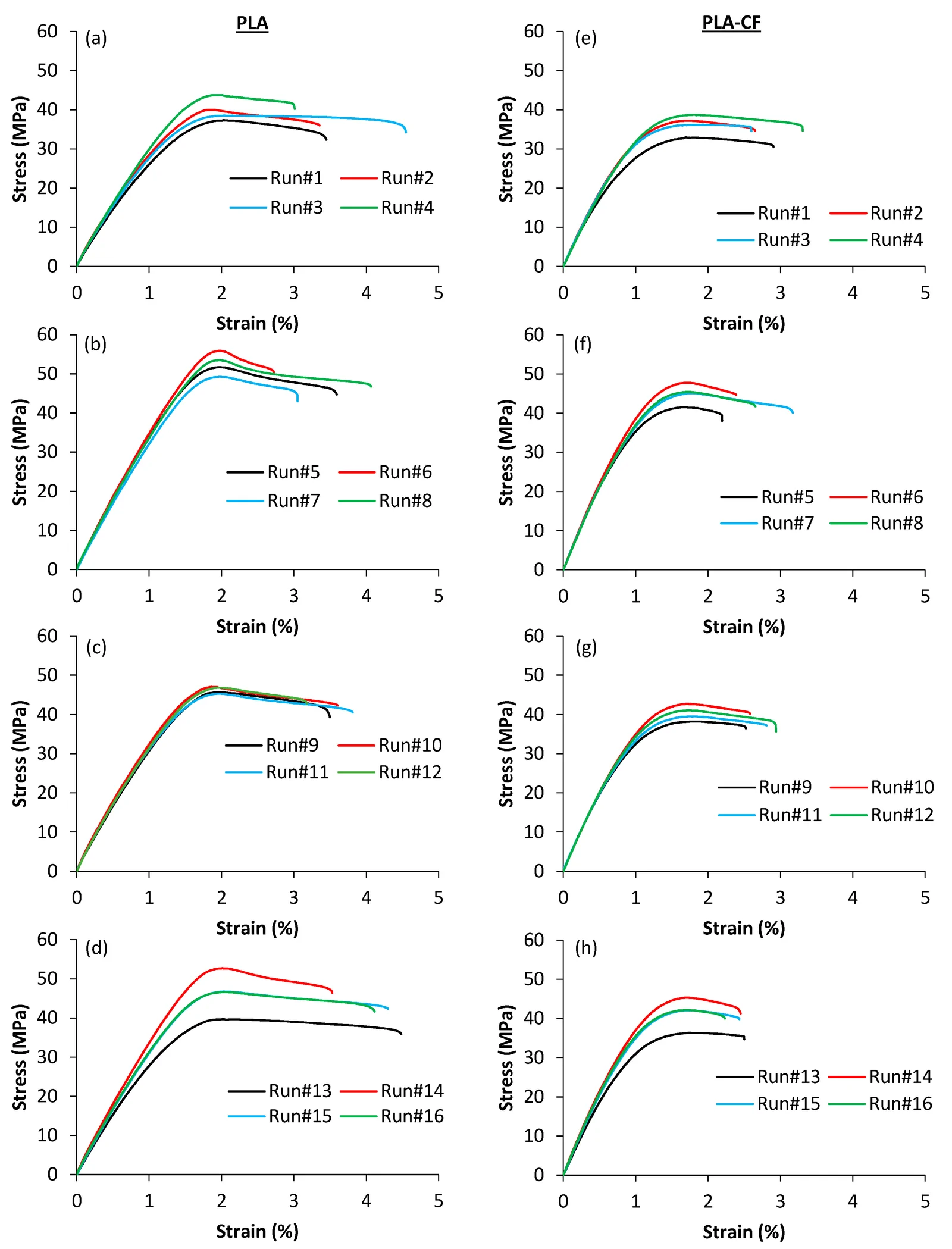
Typical stress–strain curves of the experimental runs listed in Table 4 of PLA (**a**–**d**) and PLA-CF (**e**–**h**): (**a**,**e**) run#1–run#4, (**b**,**f**) run#5–run#8, (**c**,**g**) run#9–run#12, and (**d**,**h**) run#13–run#16.

Table 4 presents the average values of the elastic modulus (E), yield strength (Yield), ultimate tensile strength (UTS), and weights obtained from the 20 experimental runs designed using the RSM for both materials, PLA and PLA-CF. The variability among the three repeated experimental results for the measured responses is low, as reflected by the standard deviation (SD) in Table 4. This indicates high repeatability in the FDM printing process. The tensile results, including E, Yield, and UTS, were obtained from the stress–strain curves. Figure 5 presents the typical stress–strain curves obtained from the RSM experimental runs for PLA and PLA-CF materials listed in Table 4.

### 3.1. ANOVA Analysis

The statistical analysis of the considered FDM printing parameters and their influence on E, Yield, UTS, and weight was conducted using ANOVA. The different fitting characteristics for the considered responses are presented in Table 5, which illustrates that the considered FDM printing parameters (ET, LT, and EM) explain most of the variability in the responses, as evidenced by the high R^2^ values exceeding 97% for all responses. Table 5 also demonstrates good predictive capability of the developed models, with predicted R^2^ values exceeding 90% across all considered responses. The consistency between the adjusted and predicted (R^2^) values provides further evidence of satisfactory model performance, with their differences remaining within the generally accepted 20% criterion. This indicates that the fitted models have reasonable predictive ability without substantial overfitting. Moreover, the adequate precision values exceeded 4 for all responses, demonstrating that the model signals were sufficiently strong relative to the associated noise and that the models were appropriate for exploring the experimental design space. The normality assumption was satisfied, as all corresponding *p*-values exceeded 0.05 (see Table 5).

#### 3.1.1. ANOVA Analysis of PLA Material

Table 6 presents the reduced ANOVA tables of E, Yield, UTS, and weights for PLA material. The ANOVA tables indicate that the developed models are statistically significant (*p* = 0.000) and account for most of the variability in the responses, as evidenced by their high contribution. Table 6 also shows the contribution percentage of each term included in the reduced ANOVA tables.

Table 6 shows that the FDM printing factors, including ET and EM, had a significant effect on E, whereas LT showed no significant effect. It also indicates that EM is the most significant FDM printing factor affecting E, accounting for 87.71% of the total variation. The interaction between LT and EM had a significant effect on E and was the second most influential term, contributing 6.29% to the total variation. ET has a relatively small but significant contribution (3.74%). The quadratic effect of EM (EM^2^) is statistically significant and accounts for 0.77% of the variance, indicating a slight nonlinear effect of EM. Overall, the statistical results show that EM is the dominant factor influencing the E, followed by the interaction between LT and EM, while LT has no significant effect.

For the yield strength response, Table 6 shows that all considered factors, including ET, LT, and EM, had a significant effect on yield strength. EM is the most significant factor affecting yield strength, accounting for 89.65% of the total variation, followed by ET, the second most influential factor, contributing 5.76%. The interaction between LT and EM had a significant effect on yield strength, contributing 2.10%. Although LT is statistically significant, it shows a relatively small contribution (0.61%), suggesting a measurable but limited influence on yield strength. Overall, the statistical findings indicate that EM is the main factor influencing yield strength, followed by ET and the LT×EM interaction.

Regarding the ultimate tensile strength, Table 6 illustrates that ET and EM had a significant effect on UTS, whereas LT showed no significant effect. EM is the most influential factor affecting UTS, accounting for 90.50% of the total variation, followed by ET, the second most influential factor, at 5.33%. The interaction between LT and EM parameters had a significant effect on UTS and contributed 2.40%. Overall, the findings indicate that EM is the dominant factor affecting the UTS response, followed by ET and the LT×EM interaction, while LT has no significant influence on the UTS.

Finally, only EM and LT had a significant effect on the sample weight, with EM being the most influential factor, accounting for 88.14% of the total variation in weight, followed by LT at 11.34%.

**Figure 6:**
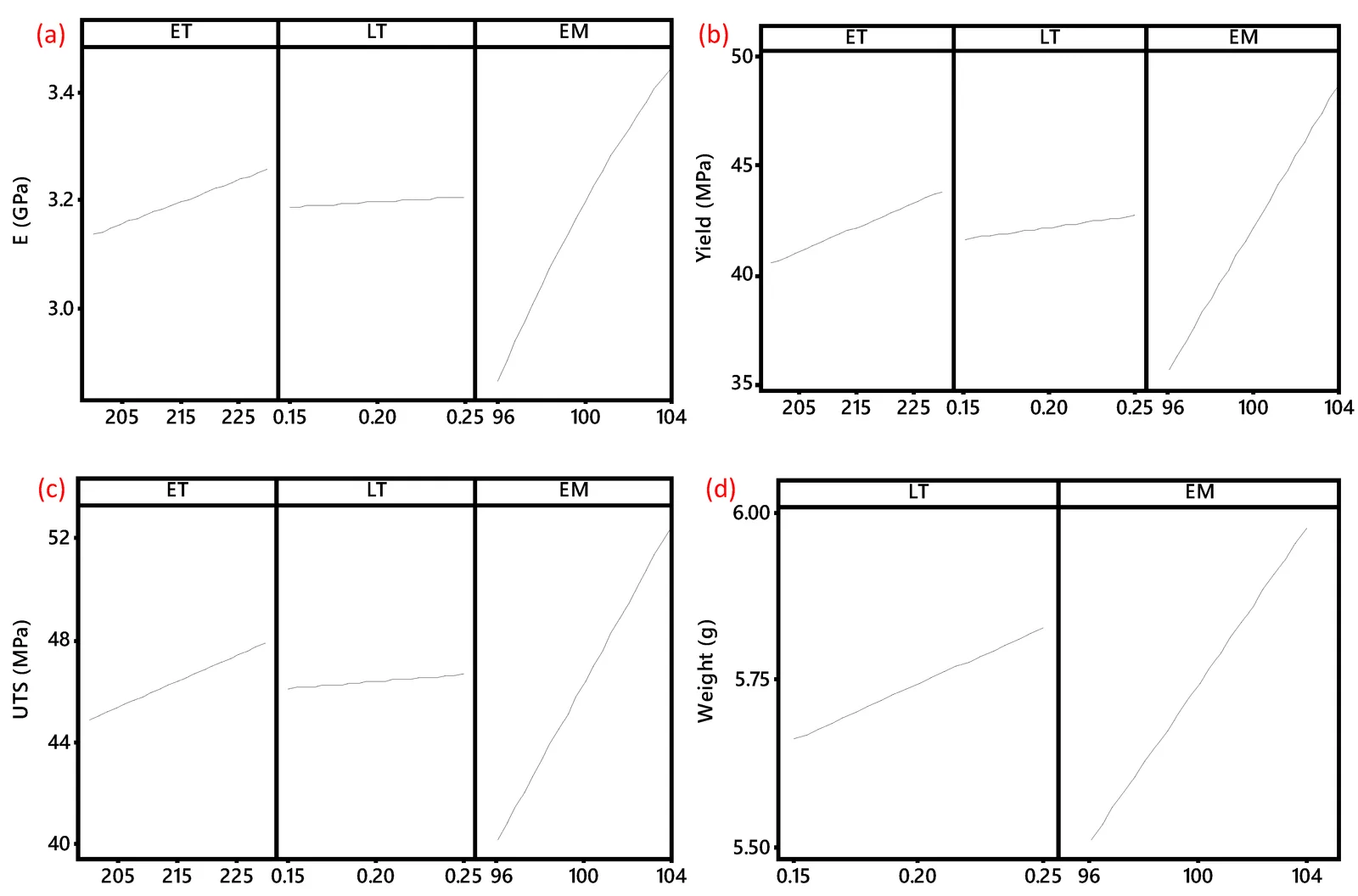
Main effect plots showing the effects of the FDM process parameters on the properties of PLA specimens: (**a**) E, (**b**) Yield, (**c**) UTS, and (**d**) weight.

**Figure 7:**
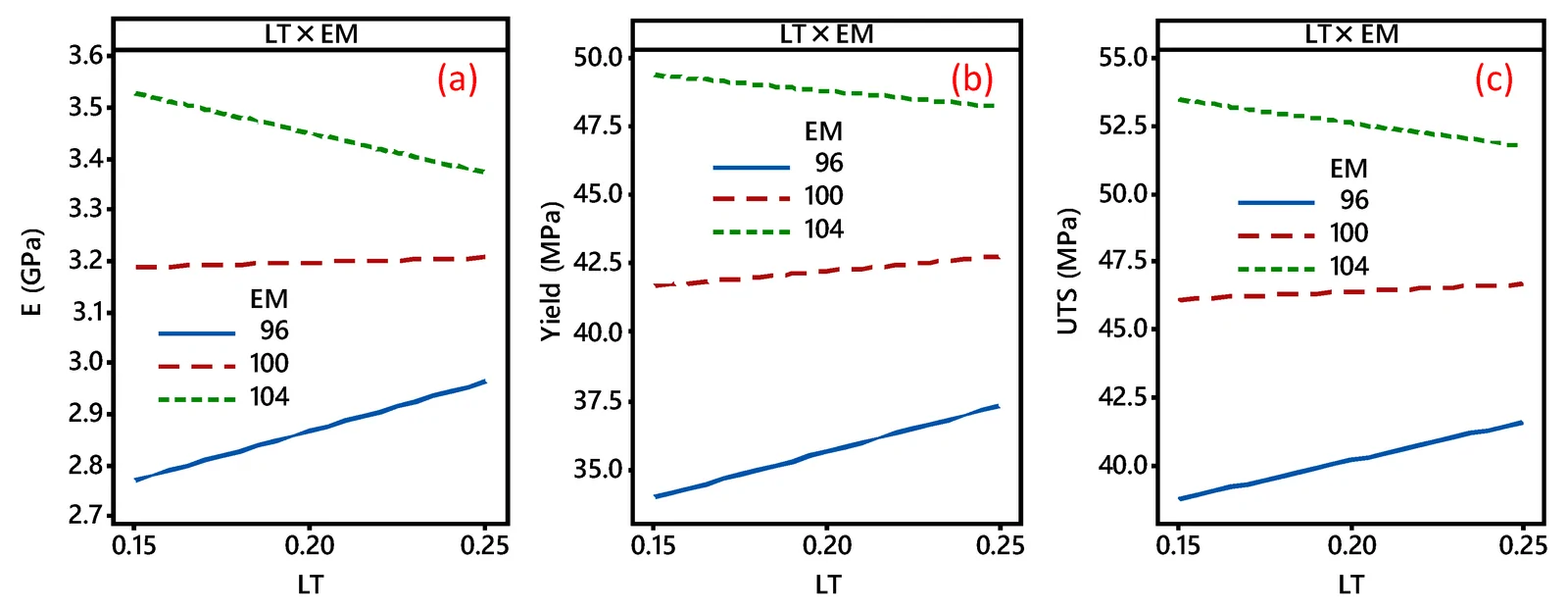
Interaction plots of FDM process parameters on the responses of PLA material: (**a**) LT and EM on E, (**b**) LT and EM on Yield, and (**c**) LT and EM on UTS.

Figure 6 presents the directional effects of the main factors (ET, LT, and EM) on E (Figure 6a), yield strength (Figure 6b), UTS (Figure 6c), and weight (Figure 6d) for PLA material. It is evident from Figure 6 that the effects of these parameters are proportional, meaning that an increase in the significant parameters (see Table 6) leads to an increase in the corresponding responses. For instance, increasing the significant factors, namely LT and EM, from their low to their high levels increases weight. Similarly, Figure 7 illustrates how the interaction between LT and EM affects E (Figure 7a), yield strength (Figure 7b), and UTS (Figure 7c) for PLA material. In general, the directional influence of the LT×EM interaction exhibits a similar pattern for E, yield strength, and UTS. For instance, an increase in LT resulted in an improvement in UTS at low EM, whereas the opposite trend was observed at high EM, where UTS decreased with increasing LT (see Figure 7c).

#### 3.1.2. ANOVA Analysis of PLA-CF Material

Table 7 presents the reduced ANOVA tables for E, Yield, UTS, and weight of the PLA-CF material. The ANOVA tables indicate that the developed models are statistically significant (*p* = 0.000) and explain most of the variability in the responses, as evidenced by the high model contribution. Table 7 also shows the contribution percentage of each term included in the reduced ANOVA tables.

Table 7 shows that the FDM printing factors, including ET and EM, had a significant effect on E of the PLA-CF material, whereas LT showed no significant effect. It also indicates that EM is the most significant FDM printing factor affecting E, contributing 84.78% of the total variation. ET contributes 3.44% of the total variation in E, while its quadratic term (ET^2^) is also significant, accounting for an additional 2.10%, indicating a nonlinear effect of ET. The interactions between ET and LT and between LT and EM are statistically significant, with contributions of 3.62% and 2.90%, respectively.

For the yield strength response, Table 7 shows that all considered factors, including ET, LT, and EM, had a significant effect on yield strength. EM is the most significant factor affecting yield strength, accounting for 75.74% of the total variation, followed by ET, the second most influential factor, at 14.19%. In addition, the quadratic effect of ET (ET^2^) is significant, contributing 1.33% and indicating a nonlinear relationship between ET and yield strength. LT contributes 1.17%, indicating a relatively small effect on the yield strength. The interactions between ET and LT and between LT and EM are statistically significant, with contributions of 2.33% and 2.17%, respectively.

Regarding the UTS of PLA-CF samples, Table 7 illustrates that ET, LT, and EM had a significant effect on UTS. It also indicates that EM is the most influential factor affecting UTS, contributing 81.05% of the total variation, followed by ET as the second most influential factor, contributing 10.66%. Also, the quadratic effect of ET (ET^2^) is significant, contributing 1.24% and indicating a slight nonlinear effect of ET on UTS. LT showed a relatively minor influence on UTS, contributing 1.01%. The interaction between ET and LT is statistically significant, with a contribution of 2.53%.

Finally, Table 7 indicates that only EM and LT had a significant effect on the weight of PLA-CF samples. Among these factors, EM was the most influential, accounting for 88.80% of the total variation in weight, followed by LT with a contribution of 10.49%.

**Figure 8:**
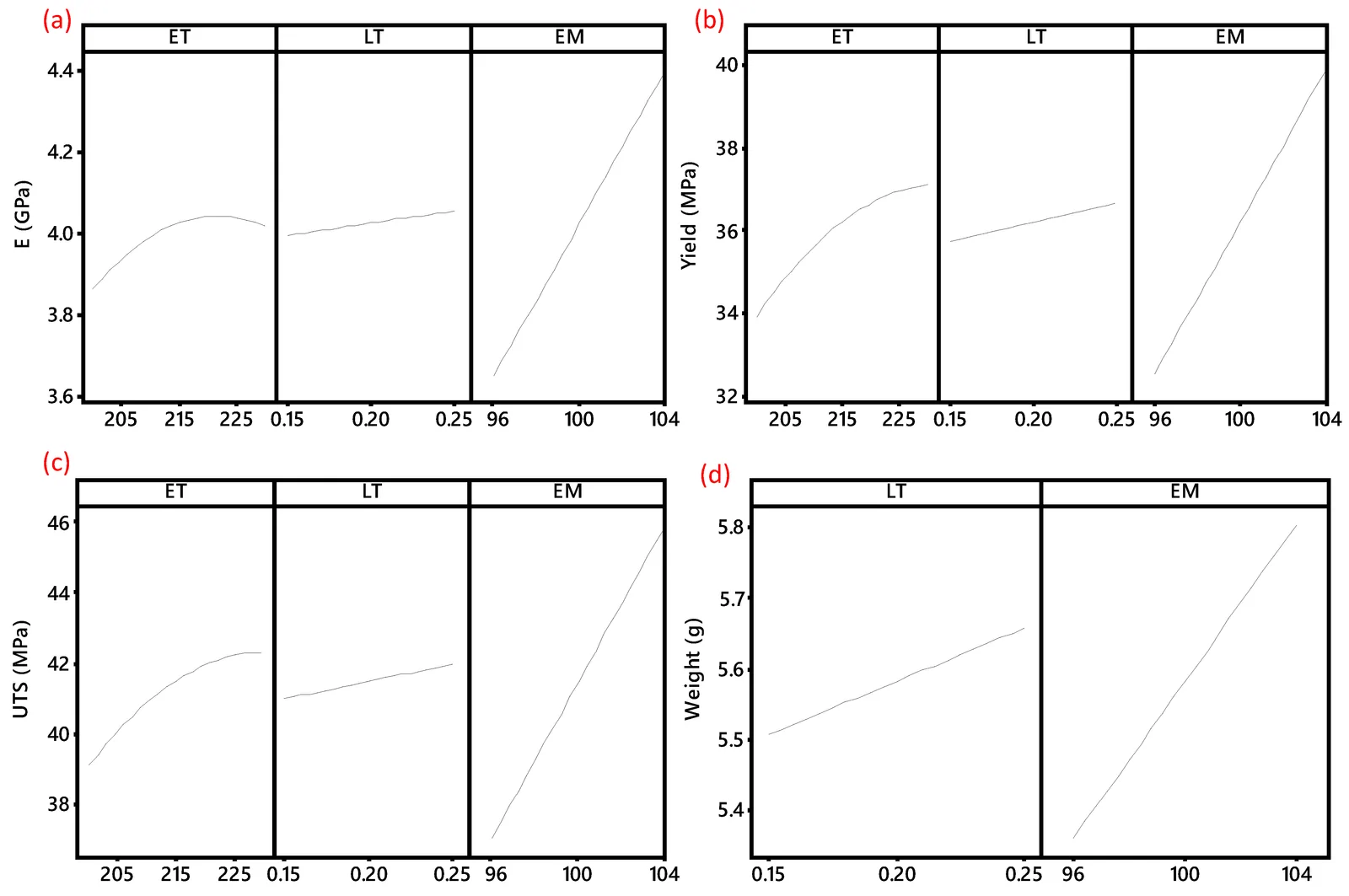
Main effect plots showing the effects of the FDM process parameters on the properties of PLA-CF specimens: (**a**) E, (**b**) Yield, (**c**) UTS, and (**d**) weight.

Figure 8 presents the directional effects of the main factors (ET, LT, and EM) on E (Figure 8a), yield strength (Figure 8b), UTS (Figure 8c), and weight (Figure 8d) for PLA-CF material. It is evident from Figure 8 that the effects of LT and EM parameters are proportional, meaning that increases in these parameters lead to increases in the corresponding responses. For instance, increasing the significant factors affecting weight, namely LT and EM, as reported in Table 7, from their low levels to their high levels, leads to increased weight. Regarding ET, Figure 8a indicates that E increases with increasing ET, followed by a slight decline at the maximum ET.

**Figure 9:**
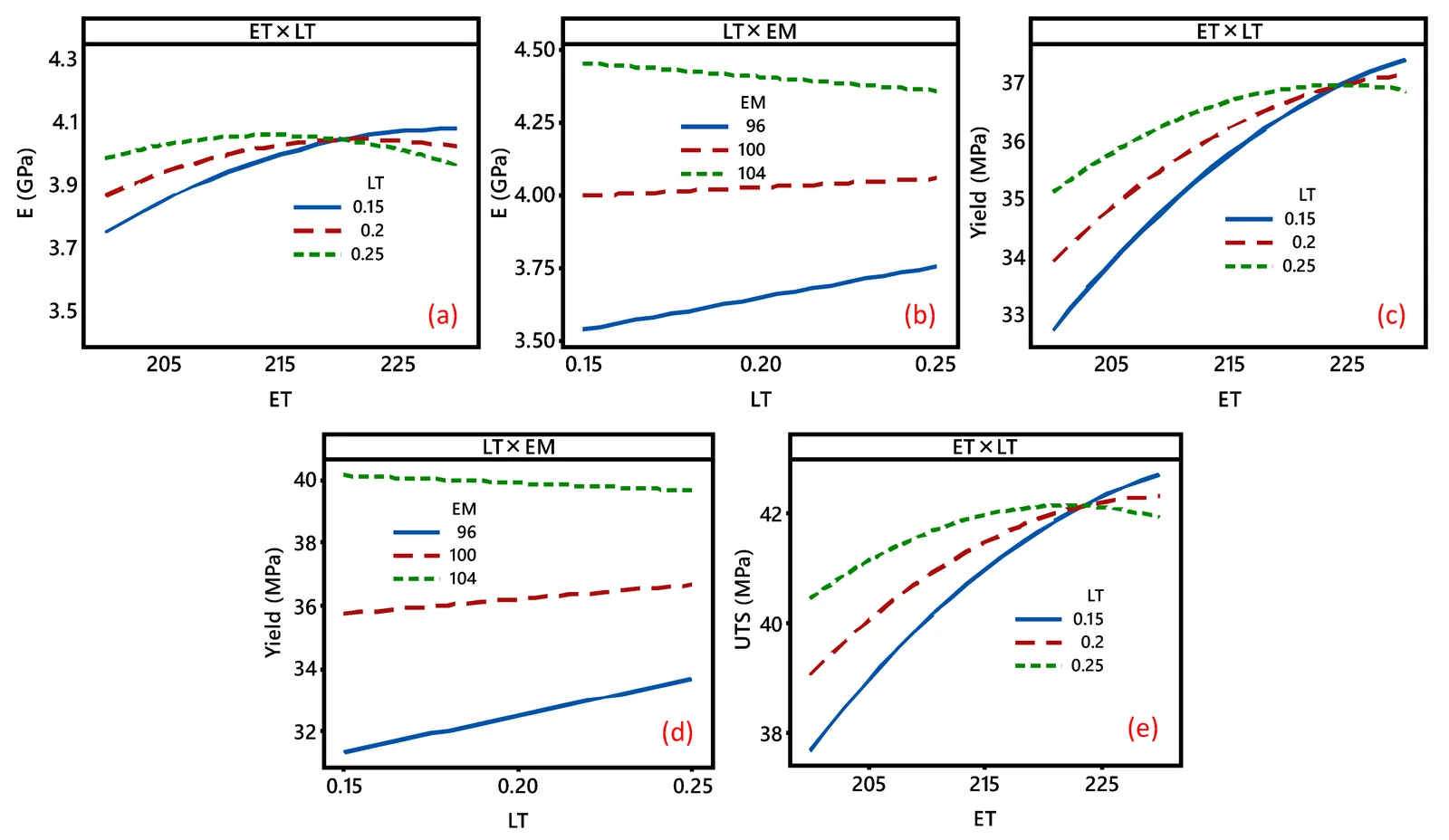
Interaction plots of FDM process parameters on the responses of PLA-CF material: (**a**) ET and LT on E, (**b**) LT and EM on E, (**c**) ET and LT on Yield, (**d**) LT and EM on Yield, and (**e**) ET and LT on UTS.

Similarly, Figure 9 illustrates how the interactions between the FDM parameters affect E, yield strength, and UTS in PLA-CF material. The directional influence of the ET×LT interaction exhibits a similar pattern for E (Figure 9a), yield strength (Figure 9c), and UTS (Figure 9e). For instance, an increase in ET resulted in an improvement in E at low LT, whereas the opposite trend was observed at high LT, where E slightly decreased with further increases in ET (see Figure 9a). Similarly, the directional influence of the LT×EM interaction exhibits a similar pattern for E (Figure 9b) and yield strength (Figure 9d). For example, an increase in LT resulted in an improvement in E at low EM, whereas the opposite trend was observed at high EM, where E decreased with increasing LT (see Figure 9b).

### 3.2. Prediction Modeling

Table 8 presents the mathematical modeling of the responses, E, Yield, UTS, and weight for both materials, PLA and PLA-CF. The mathematical models are developed based on the reduced quadratic models presented in the ANOVA tables reported in Table 6 and Table 7.

### 3.3. Multi-Objective Optimization

The optimal combination of the considered FDM parameters, namely ET, LT, and EM, was determined using desirability analysis to obtain the best overall compromise between tensile performance (E, Yield, and UTS) and material consumption, with all four responses assigned equal importance. As illustrated in Table 9, the optimal settings of 230 °C ET, 0.15 mm LT, and 104% EM achieved overall desirability values of 71.77% and 72.11% for PLA and PLA-CF, respectively. The predicted and experimental results showed close agreement, as evidenced by the small discrepancies between the predicted and experimental values, with a maximum percentage error of −1.35%, as presented in Table 9.

## 4. Discussion

The results reported in Table 4 showed that, for PLA and PLA-CF, the best tensile properties, namely E, yield strength, and UTS, were obtained in run #6 at 230 °C ET, 0.15 mm LT, and 104% EM. This parameter combination resulted in the maximum E (3.585 GPa), yield strength (51.23 MPa), and UTS (55.13 MPa) for PLA samples. Similarly, this combination yielded the maximum E (4.507 GPa), yield strength (41.30 MPa), and UTS (47.57 MPa) for PLA-CF samples. In contrast, the lowest tensile properties in both materials were obtained in run #1 at low ET (200 °C), LT (0.15 mm), and EM (96%). As shown in Table 4, run#1 resulted in the minimum E (2.68 GPa), yield strength (33.13 MPa), and UTS (37.77 MPa) for PLA samples. Similarly, the minimum E (3.233 GPa), yield strength (28.33 MPa), and UTS (33.10 MPa) were obtained for PLA-CF samples.

The difference between runs #1 and #6 is primarily attributed to the ET and EM settings. In run #1, the combination of lower EM (96%) and lower ET (200 °C) promotes the formation of voids and gaps between rasters, weakening intralayer and interlayer bonding and thereby reducing mechanical performance. For instance, the deposited material weights in run #1 were 5.429 g and 5.298 g for PLA and PLA-CF, respectively, representing the lowest weight for PLA and the second lowest for PLA-CF among the 20 RSM runs (the lowest PLA-CF weight was 5.281 g in run #2). In contrast, the higher EM (104%) and ET (230 °C) applied in run #6 enhance material deposition and bonding quality, resulting in improved mechanical performance. Correspondingly, the deposited material weights in run#6 increased to 5.907 g and 5.728 g for PLA and PLA-CF, respectively. It should be noted that an increase in sample weight does not necessarily indicate a proportional improvement in mechanical performance. For instance, although the maximum material deposition was observed in runs #7 and #8, the best mechanical properties were achieved in run #6, which exhibited lower deposited material than runs #7 and #8 (see Table 4). While EM directly governs material deposition, the role of ET in enhancing mechanical performance does not necessarily imply an increase in material weight. Instead, higher ET facilitates material flow, enabling better coverage of gaps and voids [22] and consequently strengthening interlayer and intralayer bonding. The results presented in Table 4, together with the ANOVA tables in Table 6 and Table 7, indicate that increasing ET has no effect on the amount of extruded material. For instance, a comparison of the specimens printed at 200 °C and 230 °C under different LT and EM conditions (runs #1–10 in Table 4) indicates that increasing ET from 200 °C to 230 °C has no noticeable effect on the corresponding specimen weight for either material. However, increasing ET generally enhances the mechanical properties of the materials. For instance, comparing runs #1 and #2, the E, yield strength, and UTS of PLA improved by 6.98%, 6.85%, and 6.17%, respectively. Similarly, for PLA-CF, the corresponding improvements were 14.04%, 17.30%, and 14.11%, respectively. This suggests that, for the same weight, mechanical properties can be improved by increasing the extrusion temperature under the conditions considered. It should be noted that the effect of increasing ET on the mechanical performance of PLA samples was obvious under different LT and EM settings. However, the effect of increasing ET for PLA-CF samples was less pronounced at higher LT and EM levels, as observed in runs #7 and #8. This may be attributed to the reinforcing effect of carbon fibers, which limits further mechanical gains from improved interlayer bonding at higher extrusion temperatures. Interestingly, although runs #7 and #8 yielded the maximum sample weights for both PLA and PLA-CF, this increase in material deposition did not translate into a proportional improvement in mechanical performance.

**Figure 10:**
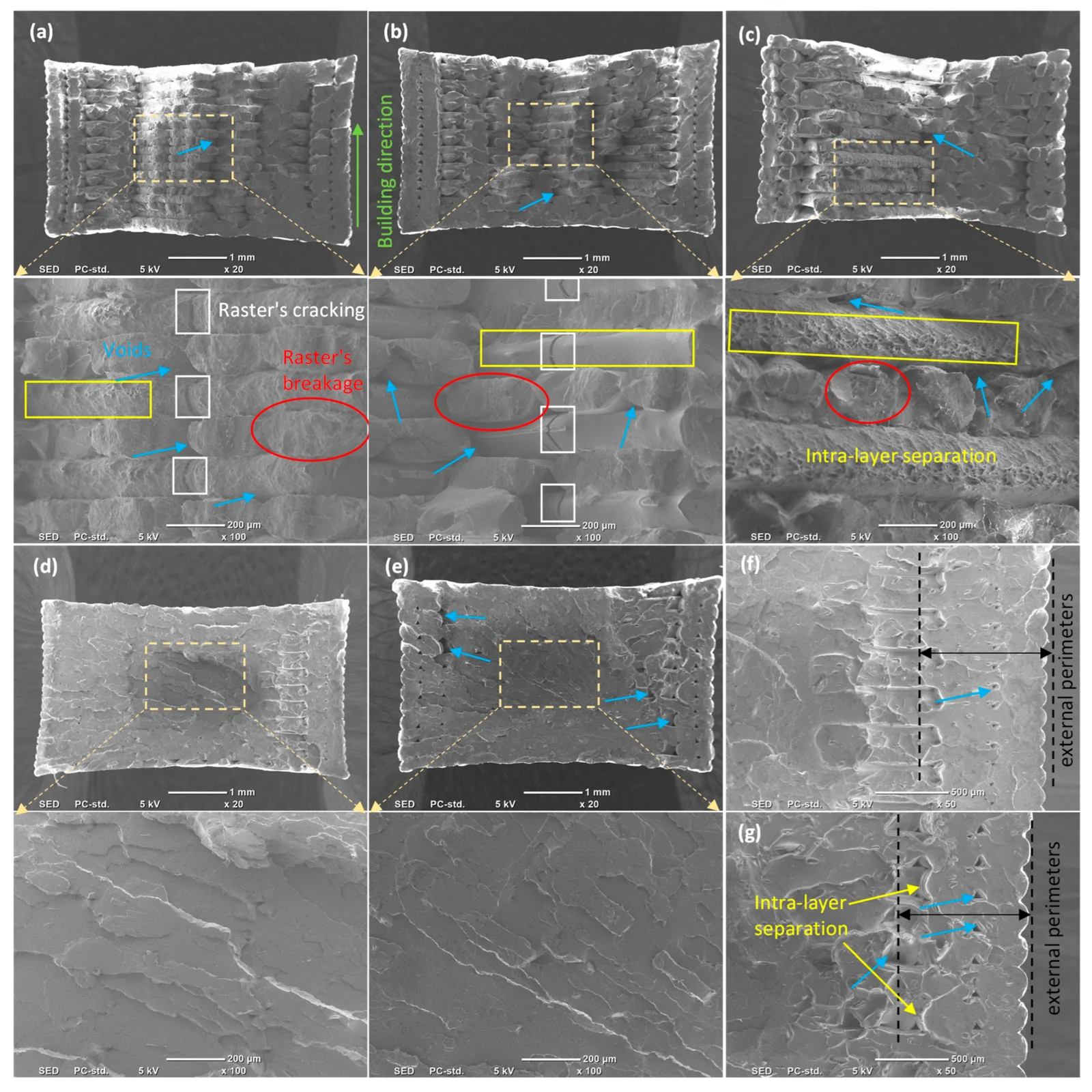
SEM images depicting the fractography of the PLA tensile-tested samples: (**a**) run#1, (**b**) run#2, (**c**) run#3, (**d**) run#6, (**e**) run#8, (**f**) run#6, and (**g**) run#8.

**Figure 11:**
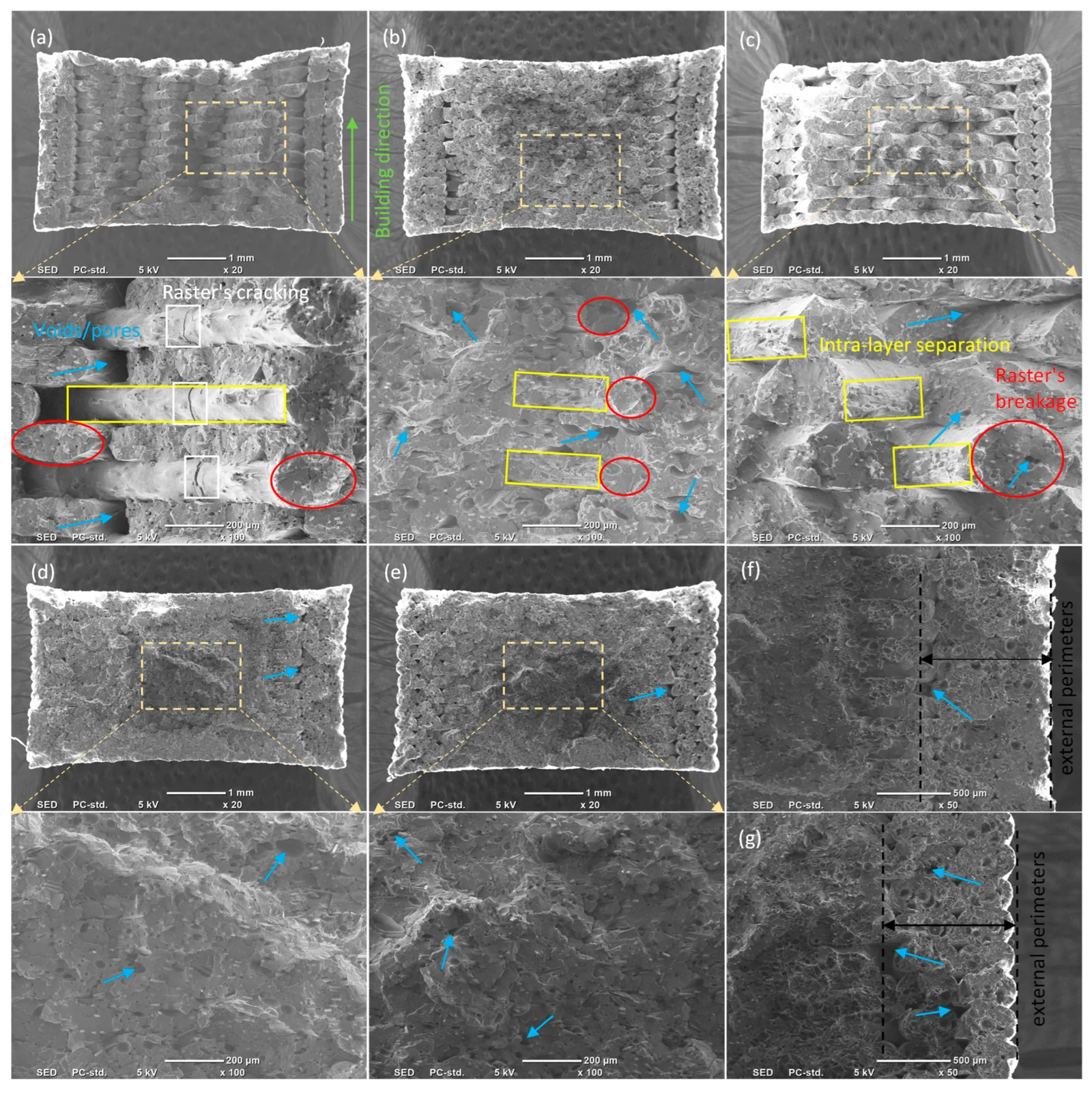
SEM images depicting the fractography of the PLA-CF tensile-tested samples: (**a**) run#1, (**b**) run#2, (**c**) run#3, (**d**) run#6, (**e**) run#8, (**f**) run#6, and (**g**) run#8.

To further investigate the influence of the considered FDM parameters on tensile performance, SEM images of fractured tensile samples were analyzed to assess the corresponding fractographic features and fracture mechanisms. Figure 10 and Figure 11 present the fracture morphologies of PLA and PLA-CF, respectively, for tensile-tested samples corresponding to RSM runs #1, #2, #3, #6, and #8 listed in Table 4. Figure 10a–e shows the PLA samples, while Figure 11a–e shows the corresponding PLA-CF samples for the same runs.

Figure 10a–c compares the cross-sectional fracture morphology of samples printed at the minimum EM (96%) under different ET and LT conditions, including low ET (run #1, Figure 10a) versus high ET (run #2, Figure 10b), and low LT (run #1, Figure 10a) versus high LT (run #3, Figure 10c). Figure 10a–c exhibit similar behavior, with a raster-dominated/interfacial fracture morphology evidenced by raster fractures and intra-layer separation. Inter-layer separation (highlighted in yellow) is mainly associated with the raster orientation used during FDM printing, which is ±45° in this work. The SEM fractography in Figure 10a–c are characterized by clearly visible individual extruded rasters, highly distinct layer interface boundaries, and the prevalent presence of voids, pores, gaps, and intra-raster separation, indicating weak interfacial adhesion. These features suggest that failure occurred along the print boundaries rather than through the bulk material. Under tensile loading, the voids, gaps, and pores acted as severe stress concentrators, allowing cracks to propagate along the weak, poorly bonded polymer interfaces and causing clean delamination before the bulk material could undergo significant plastic deformation. As illustrated, these samples exhibit similar failure mechanisms, indicating that, regardless of the ET and LT settings, the EM governs the failure mechanism. Figure 10 also shows that, at 96% EM, rasters printed with a lower LT (0.15 mm) in run #1 (Figure 10a) and run #2 (Figure 10b) were more susceptible to cracking and fracturing during tensile loading (highlighted in white) than those printed with a higher LT (0.25 mm), as observed in run #3 (Figure 10c). This could be attributed to the fact that, at lower EM, interfacial bonding is weak and load resistance is primarily carried by the rasters themselves. Thus, at higher LT, the increased raster diameter increases load-bearing capacity, resulting in higher resistance to deformation and fracture.

Figure 10d,e shows the fracture morphology of samples FDM-printed at the maximum EM (104%) and higher ET (230 °C), comparing low LT (run #6, Figure 10d) with high LT (run #8, Figure 10e). Unlike the failure pattern observed in Figure 10a–c, the samples corresponding to run #6 and run #8 (Figure 10d and Figure 10e, respectively) exhibit different failure mechanisms. The fractographic features reveal the absence of distinct raster boundaries and macro-porosity, particularly within the infill cross-section, resulting in a near-solid bulk structure. The fracture morphology suggests that the fracture occurred through the bulk material within the polymer itself rather than along raster interfaces. The presence of failure across multiple layers implies that the behavior is dominated by bulk material properties rather than by layer bonding, as also highlighted by [15]. Although run#6 and run#8 (Figure 10d and Figure 10e, respectively) exhibit similar failure mechanisms, differences are observed in pore formation at the external perimeters and at the infill-perimeter interface. For instance, the presence and size of pores/voids are less prominent at lower LT (run#6, Figure 10f) than at higher LT (run#8, Figure 10g). Also, the raster boundaries are more visible at higher LT Figure 10g than at lower LT Figure 10f. The reduction in voids increases the bonding area between printed rasters, thereby enhancing strength properties [15]. This behavior is consistent with the enhanced tensile properties (E, yield strength, and UTS) observed in run#6 compared to run#8, as reported in Table 4.

**Figure 12:**
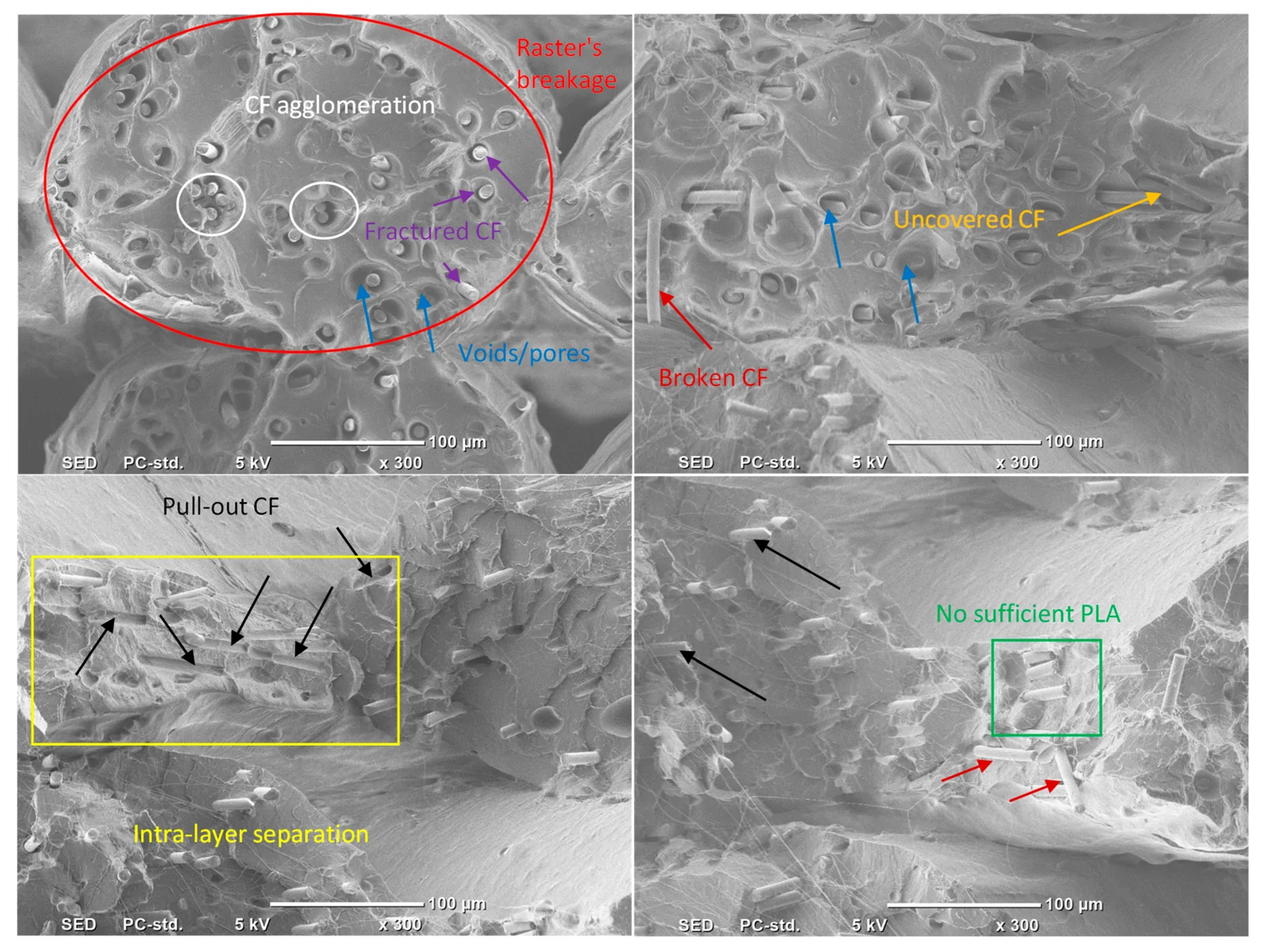
SEM morphologies showing CF-related failure features in PLA-CF samples.

Generally, the PLA-CF samples (Figure 11) exhibited fracture behavior similar to that of the PLA samples (Figure 10). However, pores and voids were more prevalent in the PLA-CF samples than in neat PLA, even at the higher EM level (104%) (Figure 11d–f). Unlike the more continuous fracture surface of PLA at high EM (Figure 10d,e), PLA-CF showed irregular morphologies with pores/voids and localized discontinuities (Figure 11d,e), likely due to CF incorporation. High-magnification SEM morphologies presented in Figure 12 reveal different failure mechanisms associated with CF addition, including void/pore formation, CF agglomeration, CF pull-out and fracture, and reduced matrix deposition at the raster-to-raster interface, as highlighted in different colors. These features contribute to the failure of the composite material, i.e., PLA-CF. Although these features may have little effect during the elastic stage, they become more influential as the stress approaches the yield point, acting as crack initiation sites and facilitating crack propagation. Consequently, the adverse effects of these failure mechanisms are primarily reflected in the reduction in the yield strength and UTS rather than the elastic modulus. Nevertheless, the reinforcing effect of CF incorporation is evident from the consistently higher stiffness (E) of the PLA-CF samples compared with neat PLA across all 20 RSM runs listed in Table 4.

The statistical analysis showed that EM had a significant effect on all responses among the considered FDM parameters, exhibiting the strongest influence on both tensile properties and material consumption. This observation is further supported by the fracture morphologies and failure mechanisms presented in Figure 10 and Figure 11. For example, at a lower EM (96%) (PLA: Figure 10a–c; PLA-CF: Figure 11a–c), pores and interlayer separation were the dominant fracture features. In contrast, at a higher EM (104%), the absence of distinct raster boundaries and macro-porosity was observed, while fractures occurred through the bulk polymer, indicating improved raster-to-raster bonding. ET also had a significant effect on all tensile properties and ranked second after EM in terms of influence. In general, the extent to which EM and ET affected the tensile properties of FDM-printed pure PLA and PLA-CF differed. Although ET significantly influenced the tensile properties of both materials, its contribution was more pronounced in the composite material (PLA-CF) than in pure PLA. For instance, the significant contributions of ET and ET^2^ to the E, yield strength, and UTS of the PLA-CF samples were 5.54%, 15.52%, and 11.9%, respectively, as shown in Table 7. In contrast, the significant contributions of ET to the E, yield strength, and UTS of the PLA samples were 3.74%, 5.76%, and 5.33%, respectively, as shown in Table 6. This suggests that EM, which directly governs the amount of deposited material, plays a more dominant role in determining the tensile properties of pure PLA than of PLA-CF, despite its high contribution to the responses in both materials, ranging from 75.47% to 90.50%. In contrast, ET exhibited the opposite trend, showing a greater influence on the composite material (PLA-CF) than on the pure PLA samples. The greater influence of EM on the tensile properties of pure PLA, compared with the composite material, could be attributed to its effect on the amount of deposited material. This can also be linked to the weight results presented in Table 4, which showed that the PLA samples consistently weighed more than the PLA-CF samples across all 20 RSM experiments. The lower weight of the PLA-CF samples can be attributed to their higher viscosity, which increases material flow resistance during deposition and generates an opposing pressure against the extrusion flow, resulting in less deposited material compared with PLA. Interestingly, the interaction between EM and ET exhibited a similar trend, showing a significant influence on the E, yield strength, and UTS of the PLA-CF material, while having no significant effect on pure PLA. LT exhibited the smallest effect on the tensile properties, with the statistical analysis confirming its influence only on the yield strength and UTS of PLA-CF, and on the yield strength of PLA, with marginal contributions. However, the interaction between LT and EM significantly affected the tensile properties, except for the UTS of PLA-CF. The ANOVA analysis further revealed that only EM and LT significantly affected the samples’ weights, with comparable contributions for both materials. Specifically, EM and LT contributed 88.14% and 11.34%, respectively, to the total weight variation in PLA samples, while the corresponding contributions for PLA-CF were 88.80% and 10.49%, respectively. These findings suggest that both parameters (EM and LT) play a substantial role in influencing the PLA and PLA-CF samples’ weight, with EM being the dominant contributor.

It is evident from the ANOVA analysis of the RSM results that EM has the greatest influence on the tensile properties (E, yield strength, and UTS) and the samples’ weight, with a proportional effect: increasing EM enhances tensile performance and increases the samples’ weight. Accordingly, based on these findings, a sensitivity analysis was subsequently conducted to examine how further increases in EM beyond the investigated RSM range affect the tensile properties and sample weight. To do so, the optimal FDM printing parameter settings, particularly ET and LT, reported in Section 3.3 and Table 9, were selected as the reference conditions for the sensitivity analysis. Thus, the optimum ET and LT settings reported in Table 9 were maintained (ET = 230 °C and LT = 0.15 mm), while EM was further increased to 106%, 110%, and 115%, which were outside the range of the original RSM design. Table 10 presents the observed changes in the tensile properties and sample weight as EM was increased to 106%, 110%, and 115%. Table 10 shows that at 106% EM, the tensile properties of PLA samples are comparable to those at the optimum condition (i.e., 104% EM). However, further increasing the EM considerably degrades tensile properties, even with a proportional increase in sample weight. This trend is consistent with the findings of Lendvai et al. [12], who reported that the mechanical properties of FDM-printed PLA parts improved up to an EM of 103%, whereas further increasing the EM to 105% resulted in a decrease in Charpy impact strength and flexural strength due to excessive material compression, accompanied by reduced geometrical precision. Similarly, Lambiase et al. [14] reported that PLA specimens printed at 104% EM exhibited lower fracture toughness than those printed at 100% EM, which was attributed to poor adhesion resulting from increased material flow. Regarding the PLA-CF samples, the tensile properties are enhanced at EM values of 106% and 110% compared to the optimum condition (104%), with no noticeable difference between the results obtained at 106% and 110%. For instance, an increase in the EM from 104% to 106% improved E, yield strength, and UTS of PLA-CF material by 9.41%, 6.03%, and 5.65%, respectively. However, a further increase in EM above 110%, i.e., at 115%, results in a decline in the tensile properties of the PLA-CF samples.

It should be noted that at an EM above 106%, printing quality deteriorated due to over-extrusion, resulting in excessive material accumulation on the top surfaces, as evidenced by visible ridges, and consequently higher surface roughness. Furthermore, over-extrusion at an EM of 115% causes excessive material accumulation between adjacent rasters, forming pronounced surface ridges that generate friction between the nozzle and the previously deposited layer, as evidenced by the frictional noise during FDM printing. In addition, significant deviations in the dimensions of the tensile samples from the designed geometry are observed. For instance, the thickness of the PLA tensile samples at 104% EM (i.e., optimal condition), 106%, 110%, and 115% are 3.182 mm, 3.25 mm, 3.40 mm, and 3.55 mm, respectively. Consequently, printability issues, such as surface quality and dimensional accuracy, become more severe at EM levels above 106%, making these EM levels unsuitable for FDM printing of PLA and PLA-CF materials. Thus, based on the sensitivity analysis, increasing the EM from 104% to 106% enhanced the tensile properties of the PLA-CF samples, whereas no further increase in EM was required to enhance the tensile properties of the PLA samples.

## 5. Conclusions

This study investigated the influence of extrusion-related FDM process parameters, including ET, LT, and EM, on tensile properties and material consumption of PLA and PLA-CF. The following conclusions can be drawn from this study:

- Variations in ET, LT, and EM significantly affected the tensile properties and weight of PLA and PLA-CF. For neat PLA, E, yield, UTS, and weight varied from 2.68 to 3.583 GPa, 33.13 to 51.23 MPa, 37.77 to 55.13 MPa, and 5.429 to 6.08 g, respectively. Similarly, PLA-CF exhibited variations of 3.233–4.507 GPa in E, 28.33–41.30 MPa in yield strength, 33.1–47.57 MPa in UTS, and 5.281–5.894 g in weight.
- EM was the most influential FDM parameter, accounting for 75.47–90.50% of the total variation in E, yield strength, UTS, and samples’ weight, for both PLA and PLA-CF materials. Its influence on the tensile properties was more pronounced in the neat PLA than in the composite material (PLA-CF).
- Increasing EM shifted the failure mechanism from raster-dominated/interfacial fracture, characterized by raster fracture and interlayer separation at low EM (96%), toward bulk-material-dominated fracture at higher EM (104%). Lower EM promoted the formation of voids, pores, and inter-raster gaps, resulting in weak interfacial bonding and consequently deteriorating the mechanical properties of the FDM-printed parts.
- ET was the second most influential parameter after EM, significantly affecting all tensile properties. Increasing ET generally enhanced the tensile properties by improving the coverage of gaps and voids, thereby strengthening interfacial bonding. Its effect was more pronounced in PLA-CF than in neat PLA. For example, the contributions of ET and ET^2^ to E, yield strength, and UTS were 5.54%, 15.52%, and 11.90%, respectively, for PLA-CF, compared with 3.74%, 5.76%, and 5.33% for neat PLA.
- The interaction between EM and ET significantly influenced the E, yield strength, and UTS of PLA-CF, while showing no significant effect on neat PLA.
- LT exhibited the least influence on the tensile properties among the investigated FDM process parameters, contributing only 0.61–1.17% to the total variation.
- Only EM and LT significantly affected specimen weight, with EM being the dominant factor, for PLA and PLA-CF. For PLA, EM and LT accounted for 88.14% and 11.34% of the total weight variation, respectively, while the corresponding contributions for PLA-CF were 88.80% and 10.49%. Under identical printing conditions, PLA specimens consistently exhibited higher weights than the corresponding PLA-CF specimens.
- The reinforcing effect of CF incorporation is obvious from the consistently higher E of the PLA-CF samples compared with neat PLA.
- SEM morphologies revealed CF-associated defects, including void and pore formation, CF agglomeration, CF pull-out and breakage, and reduced matrix deposition at raster interfaces, contributing to PLA-CF failure and consequently lower yield strength and UTS than neat PLA. Moreover, the higher prevalence of pores and voids in the PLA-CF samples than in neat PLA, even at the higher EM level (104%), highlights the importance of optimizing the FDM process parameters, particularly for composite materials, to minimize these defects and improve mechanical performance.
- Using the desirability approach, the optimal settings (230 °C ET, 0.15 mm LT, and 104% EM) simultaneously maximized E (PLA: 3.583 GPa; PLA-CF: 4.507 GPa), yield (PLA: 51.23 MPa; PLA-CF: 41.30 MPa), and UTS (PLA: 55.13 MPa; PLA-CF: 47.57 MPa), while minimizing specimen weight (PLA: 5.907 g; PLA-CF: 5.728 g). Experimental validation showed excellent agreement with the predictions, with a maximum absolute error of 1.35% across all responses.
- Sensitivity analysis showed that a small increase in EM from 104% to 106% improved the tensile properties of PLA-CF, whereas 104% remained the optimal EM for PLA, with no additional improvement in tensile properties at higher EM values. These findings suggest that composite materials (e.g., PLA-CF) require a higher EM than the corresponding neat polymer (i.e., PLA). EM values above 106% degrade the surface quality and dimensional accuracy of FDM-printed parts without providing additional improvements in tensile properties.
- Overall, this study advances the understanding of the structure-processing-property relationships in FDM by providing new insights into how key process parameters influence the microstructure, mechanical performance, and material efficiency of both neat and composite polymer materials.

## Figures and Tables

**Table 1:** PLA and PLA-CF material characteristics.

Characteristic	PLA [19]	PLA-CF [20]
Density, g/cm^3^	1.24	1.29
Tensile modulus, GPa	2.3	4.95
Tensile strength at break, MPa	-	48
Tensile yield strength, MPa	51	-
Flexural strength, MPa	83	89
Flexural modulus, GPa	3.1	6.32

**Table 2:** Measured filament diameters and thermal properties.

Characteristic	PLA	PLA-CF
Filament diameter, mm	1.749 ± 0.006	1.758 ± 0.011
T_g_, °C	66.1	67.55
T_cc_, °C	119	115.2
T_m_, °C	156.5	156.6

**Table 3:** FDM parameters and their levels.

FDM Variable	Levels		
	−1	0	1
Extrusion temperature (ET), °C	200	215	230
Layer thickness (LT), mm	0.15	0.20	0.25
Extrusion multiplier (EM)	0.96	1	1.04

**Table 4:** RSM experiments along with the results (E, Yield, UTS, and weight) for PLA and PLA-CF.

Run No.	Input Parameters			Responses: PLA Material				Responses: PLA-CF Material			
	ET (°C)	LT (mm)	EM (%)	E (GPa ± SD)	Yield (MPa ± SD)	UTS (MPa ± SD)	Weight (g ± SD)	E (GPa ± SD)	Yield (MPa ± SD)	UTS (MPa ± SD)	Weight (g ± SD)
1	200	0.15	96	2.68 ± 0.03	33.13 ± 0.49	37.77 ± 0.64	5.429 ± 0.002	3.233 ± 0.04	28.33 ± 0.15	33.1 ± 0.35	5.298 ± 0.013
2	230	0.15	96	2.867 ± 0.05	35.4 ± 0.36	40.1 ± 0.17	5.43 ± 0.002	3.687 ± 0.07	33.23 ± 0.21	37.77 ± 0.51	5.281 ± 0.014
3	200	0.25	96	2.897 ± 0.04	34.3 ± 0.2	39 ± 0.46	5.567 ± 0.002	3.67 ± 0.03	31.93 ± 0.06	36.1 ± 0.27	5.445 ± 0.007
4	230	0.25	96	3.017 ± 0.02	40.33 ± 0.06	43.83 ± 0.15	5.598 ± 0.001	3.637 ± 0.02	34.3 ± 0.1	38.67 ± 0.06	5.438 ± 0.015
5	200	0.15	104	3.517 ± 0.04	47.73 ± 1.07	52.1 ± 0.46	5.878 ± 0.003	4.267 ± 0.1	37.43 ± 1.08	42.67 ± 1.02	5.713 ± 0.017
6	230	0.15	104	3.583 ± 0.03	51.23 ± 0.21	55.13 ± 0.68	5.907 ± 0.002	4.507 ± 0.07	41.3 ± 0.44	47.57 ± 0.49	5.728 ± 0.017
7	200	0.25	104	3.317 ± 0.01	46.2 ± 0.44	49.73 ± 0.68	6.061 ± 0.001	4.253 ± 0.06	38.6 ± 0.1	45.1 ± 0.4	5.894 ± 0.013
8	230	0.25	104	3.45 ± 0.02	49.87 ± 0.55	53.43 ± 0.21	6.08 ± 0.003	4.28 ± 0.02	39.2 ± 0.5	45.05 ± 0.35	5.875 ± 0.03
9	200	0.2	100	3.153 ± 0.02	41.77 ± 0.4	46.03 ± 0.45	5.76 ± 0.003	3.9 ± 0.02	33.3 ± 0.36	38.43 ± 0.49	5.573 ± 0.002
10	230	0.2	100	3.25 ± 0.09	42.97 ± 0.58	47.17 ± 0.21	5.74 ± 0.002	3.977 ± 0.01	37.57 ± 0.25	42.53 ± 0.15	5.588 ± 0.01
11	215	0.15	100	3.173 ± 0.08	40.73 ± 0.68	45.17 ± 1.05	5.664 ± 0.002	3.89 ± 0.07	35.13 ± 0.68	40.17 ± 0.7	5.516 ± 0.009
12	215	0.25	100	3.233 ± 0.02	42.97 ± 0.59	47.07 ± 0.46	5.839 ± 0.003	4.047 ± 0.06	36 ± 0.1	41.33 ± 0.21	5.648 ± 0.017
13	215	0.2	96	2.867 ± 0.02	34.93 ± 0.49	39.87 ± 0.29	5.524 ± 0.003	3.667 ± 0.02	31.7 ± 0.2	36.37 ± 0.06	5.347 ± 0.013
14	215	0.2	104	3.383 ± 0.03	48.83 ± 0.21	52.13 ± 0.49	5.954 ± 0.003	4.377 ± 0.09	39.93 ± 0.47	46.23 ± 0.81	5.822 ± 0.027
15	215	0.2	100	3.22 ± 0.03	42.6 ± 0.62	46.87 ± 0.86	5.748 ± 0	4.083 ± 0.03	37 ± 0.46	42.4 ± 0.44	5.583 ± 0.018
16	215	0.2	100	3.197 ± 0.03	42.33 ± 0.31	46.5 ± 0.46	5.752 ± 0.001	4.113 ± 0.1	35.9 ± 0.89	41.1 ± 0.95	5.577 ± 0.015
17	215	0.2	100	3.193 ± 0.05	42.73 ± 0.45	47.2 ± 0.9	5.731 ± 0.002	4.04 ± 0.05	36.83 ± 0.25	42.07 ± 0.21	5.596 ± 0.008
18	215	0.2	100	3.213 ± 0.03	41.67 ± 0.42	45.93 ± 0.32	5.737 ± 0.001	3.99 ± 0.07	37.13 ± 0.99	42.3 ± 1.15	5.607 ± 0.017
19	215	0.2	100	3.173 ± 0.03	42.03 ± 0.32	46.3 ± 0.46	5.735 ± 0.001	4.057 ± 0.07	36.57 ± 0.29	41.97 ± 0.38	5.577 ± 0.009
20	215	0.2	100	3.157 ± 0.02	42.3 ± 0.1	46.2 ± 0.27	5.753 ± 0	3.99 ± 0.02	35.93 ± 0.5	40.87 ± 0.5	5.542 ± 0.009

**Table 5:** Fitting statistics of E, Yield, UTS, and weight for PLA and PLA-CF materials.

Material	Response	R^2^ (%)	R^2^ Pred. (%)	Adjusted R^2^ (%)	Adeq. Precision	Norm. (*p*-Value) *
PLA	E	98.59	96.41	98.09	51.29	0.556
	Yield	98.52	95.35	98.00	45.43	0.455
	UTS	98.71	97.02	98.25	49.33	0.917
	Weight	99.48	99.22	99.42	119.53	0.320
PLA-CF	E	97.38	90.56	96.17	35.92	0.386
	Yield	97.48	93.42	96.01	34.55	0.510
	UTS	97.30	90.87	96.05	35.30	0.373
	Weight	99.29	99.09	99.20	100.97	0.680

* Normality *p*-value based on Anderson-Darling test.

**Table 6:** Reduced ANOVA Tables of PLA samples.

Response	Source	DF	Contribution	Adj SS	Adj MS	F-Value	*p*-Value
E	Model	5	98.59%	0.960586	0.192117	195.67	0.000
	ET	1	3.74%	0.036401	0.036401	37.07	0.000
	LT	1	0.09%	0.000871	0.000871	0.89	0.362
	EM	1	87.71%	0.854588	0.854588	870.41	0.000
	EM^2^	1	0.77%	0.007476	0.007476	7.61	0.015
	LT×EM	1	6.29%	0.06125	0.06125	62.38	0.000
	Error	14	1.41%	0.013746	0.000982		
	Lack-of-Fit	9	1.12%	0.010886	0.00121	2.12	0.212
	Pure Error	5	0.29%	0.002859	0.000572		
	Total	19	100.00%				
Yield	Model	5	98.52%	475.314	95.063	186.78	0.000
	ET	1	5.76%	27.778	27.778	54.58	0.000
	LT	1	0.61%	2.952	2.952	5.8	0.03
	EM	1	89.65%	432.525	432.525	849.81	0.000
	ET×LT	1	0.40%	1.934	1.934	3.8	0.072
	LT×EM	1	2.10%	10.125	10.125	19.89	0.001
	Error	14	1.48%	7.126	0.509		
	Lack-of-Fit	9	1.32%	6.377	0.709	4.74	0.051
	Pure Error	5	0.16%	0.748	0.15		
	Total	19	100.00%				
UTS	Model	5	98.71%	418.824	83.765	214.95	0.000
	ET	1	5.33%	22.6	22.6	57.99	0.000
	LT	1	0.18%	0.784	0.784	2.01	0.178
	EM	1	90.50%	383.987	383.987	985.35	0.000
	ET×LT	1	0.30%	1.253	1.253	3.22	0.095
	LT×EM	1	2.40%	10.2	10.2	26.17	0.000
	Error	14	1.29%	5.456	0.39		
	Lack-of-Fit	9	1.03%	4.38	0.487	2.26	0.191
	Pure Error	5	0.25%	1.076	0.215		
	Total	19	100.00%				
Weight	Model	2	99.48%	0.614398	0.307199	1637.88	0.000
	LT	1	11.34%	0.070062	0.070062	373.55	0.000
	EM	1	88.14%	0.544336	0.544336	2902.21	0.000
	Error	17	0.52%	0.003189	0.000188		
	Lack-of-Fit	12	0.44%	0.002739	0.000228	2.54	0.156
	Pure Error	5	0.07%	0.000449	0.00009		
	Total	19	100.00%				

**Table 7:** Reduced ANOVA Tables of PLA-CF samples.

Response	Source	DF	Contribution	Adj SS	Adj MS	F-Value	*p*-Value
E	Model	6	97.38%	1.64978	0.27496	80.45	0.000
	ET	1	3.44%	0.05827	0.05827	17.05	0.001
	LT	1	0.54%	0.0092	0.0092	2.69	0.125
	EM	1	84.78%	1.43641	1.43641	420.27	0.000
	ET^2^	1	2.10%	0.03556	0.03556	10.4	0.007
	ET×LT	1	3.62%	0.06125	0.06125	17.92	0.001
	LT×EM	1	2.90%	0.04909	0.04909	14.36	0.002
	Error	13	2.62%	0.04443	0.00342		
	Lack-of-Fit	8	1.89%	0.03208	0.00401	1.62	0.308
	Pure Error	5	0.73%	0.01235	0.00247		
	Total	19	100.00%				
Yield	Model	7	97.48%	175.878	25.125	66.38	0.000
	ET	1	14.19%	25.6	25.6	67.64	0.000
	LT	1	1.17%	2.116	2.116	5.59	0.036
	EM	1	75.74%	136.653	136.653	361.04	0.000
	ET^2^	1	1.33%	2.404	2.404	6.35	0.027
	ET×LT	1	2.33%	4.205	4.205	11.11	0.006
	ET×EM	1	0.54%	0.98	0.98	2.59	0.134
	LT×EM	1	2.17%	3.92	3.92	10.36	0.007
	Error	12	2.52%	4.542	0.378		
	Lack-of-Fit	7	1.73%	3.117	0.445	1.56	0.322
	Pure Error	5	0.79%	1.425	0.285		
	Total	19	100.00%				
UTS	Model	6	97.30%	238.989	39.832	78.08	0.000
	ET	1	10.66%	26.19	26.19	51.34	0.000
	LT	1	1.01%	2.483	2.483	4.87	0.046
	EM	1	81.05%	199.065	199.065	390.21	0.000
	ET^2^	1	1.24%	3.055	3.055	5.99	0.029
	ET×LT	1	2.53%	6.213	6.213	12.18	0.004
	LT×EM	1	0.81%	1.983	1.983	3.89	0.07
	Error	13	2.70%	6.632	0.51		
	Lack-of-Fit	8	1.86%	4.564	0.57	1.38	0.376
	Pure Error	5	0.84%	2.068	0.414		
	Total	19	100.00%				
Weight	Model	2	99.29%	0.552547	0.276274	1183.96	0.000
	LT	1	10.49%	0.058359	0.058359	250.1	0.000
	EM	1	88.80%	0.494188	0.494188	2117.82	0.000
	Error	17	0.71%	0.003967	0.000233		
	Lack-of-Fit	12	0.28%	0.001545	0.000129	0.27	0.972
	Pure Error	5	0.44%	0.002422	0.000484		
	Total	19	100.00%				

**Table 8:** Mathematical prediction models of E, Yield, UTS, and weight.

Material	Response	Mathematical Model
PLA	E (GPa)	$=-37.93+0.004022\;ET+43.94\;LT+0.644\;EM-0.002417\;{EM}^{2}-0.4375\;LT\times EM$
	Yield (MPa)	=−232.6−0.0200 ET+432 LT+2.769 EM+0.656 ET×LT−5.62 LT×EM
	UTS (MPa)	=−221.4−0.0053 ET+457 LT+2.678 EM+0.528 ET×LT−5.65 LT×EM
	Weight (g)	=−0.423+1.6741 LT+0.05833 EM
PLA-CF	E (GPa)	$=-36.84+0.1896\;ET+64.9\;LT+0.1731\;EM-0.000375\;{ET}^{2}-0.1167\;ET\times LT-0.392\;LT\times EM$
	Yield (MPa)	$=-460.4+2.208\;ET+567\;LT+2.878\;EM-0.00308\;{ET}^{2}-0.967\;ET\times LT-0.00583\;ET\times EM-3.50\;LT\times EM$
	UTS (MPa)	$=-356.2+1.837\;ET+512\;LT+1.613\;EM-0.00347\;{ET}^{2}-1.175\;ET\times LT-2.49\;LT\times EM$
	Weight (g)	=−0.281+1.5279 LT+0.05558 EM

**Table 9:** Optimal FDM parameter settings for PLA and PLA-CF materials.

Material	Result	Parameters			Responses				
		ET (°C)	LT (mm)	EM (%)	E (GPa)	Yield (MPa)	UTS (MPa)	Weight (g)	Desirability (%)
PLA	Pred.	230	0.15	104	3.589	50.54	54.53	5.894	71.77
	Exp.				3.583	51.23	55.13	5.907	
	Percentage error (%)				0.17	−1.35	−1.09	−0.22	
PLA-CF	Pred.	230	0.15	104	4.532	41.43	47.66	5.729	72.11
	Exp.				4.507	41.30	47.57	5.728	
	Percentage error (%)				0.55	0.31	0.19	0.02	

**Table 10:** Effect of increasing EM beyond the optimum level on E, Yield, UTS, and weight for PLA and PLA-CF materials.

	Input Parameters			Responses: PLA Material				Responses: PLA-CF Material			
	ET (°C)	LT (mm)	EM (%)	E (GPa)	Yield (MPa)	UTS (MPa)	Weight (g)	E (GPa)	Yield (MPa)	UTS (MPa)	Weight (g)
Optimal	230	0.15	104	3.583	51.23	55.13	5.907	4.507	41.30	47.57	5.728
EM:106%	230	0.15	106	3.508	52.53	55.10	6.030	4.931	43.79	50.26	5.903
EM:110%	230	0.15	110	3.314	51.20	53.29	6.212	4.906	43.92	50.67	6.132
EM:115%	230	0.15	115	3.305	46.68	50.18	6.490	4.533	41.82	47.90	6.400

## Data Availability

The original contributions presented in this study are included in the article. Further inquiries can be directed to the corresponding author.
